# Intravitreal sirolimus for persistent, exudative age-related macular degeneration: a Pilot Study

**DOI:** 10.1186/s40942-021-00281-0

**Published:** 2021-02-16

**Authors:** Robert J. Minturn, Peter Bracha, Margaret J. Klein, Jay Chhablani, Ashley M. Harless, Raj K. Maturi

**Affiliations:** 1 Department of Ophthalmology, Indiana University School of Medicine, 10300 N Illinois St, Suite 1060, Indianapolis, IN 46290 USA; 2 Gunderson Eye Institute, Gundersen Health System, La Crosse, WI USA; 3 Department of Anesthesiology Critical Care Medicine, The Saban Research Institute At Children’s Hospital, Los Angeles, CA USA; 4 UPMC Eye Center, University of Pittsburgh, Pittsburgh, PA USA; 5 Midwest Eye Institute, Indianapolis, IN USA

**Keywords:** Sirolimus, Rapamycin, Anti-vascular endothelial growth factor, Exudative age-related macular degeneration

## Abstract

**Background and objective:**

To evaluate the safety and efficacy of intravitreal sirolimus for persistent, exudative age-related macular degeneration (AMD).

**Methods:**

This institutional review board approved, registered (NCT02357342), prospective, subject-masked, single center, randomized controlled trial in subjects with persistent, exudative Age-related macular degeneration compared intravitreal sirolimus monotherapy (every 2 months) versus monthly anti-vascular endothelial growth factor (VEGF) over six months.

**Results:**

20 subjects were randomized to each arm of the trial. Upon completion of the trial 20 patients were analyzed in the control (anti-vascular endothelial growth factor) group and 17 patients were analyzed in the treatment (sirolimus) group. On average, subjects had 33 previous anti-VEGF injections prior to entry. The primary end-point, mean central subfield thickness (CST), increased by 20 µm in the anti-vascular endothelial growth factor group and decreased by 40 µm in the sirolimus group (*p* = *0.03*). Visual acuity outcomes were similar between groups. Serious ocular adverse events in the sirolimus group included one subject each with anterior uveitis, central retinal artery occlusion and subretinal hemorrhage.

**Conclusion:**

Monotherapy with intravitreal sirolimus for subjects with persistent, exudative age-related macular degeneration appears to have a limited positive anatomic benefit. The presence of adverse events in the experimental group merits further evaluation, potentially as an adjuvant therapy.

*Trial registration* This trial was registered with the clinicaltrials.gov, NCT02357342, and was approved by the institutional review board at Advarra. Funding was provided by an investigator-initiated grant from Santen. Santen played no role in the design or implementation of this study.

## Background and objective

In the Comparison of Age-Related Macular Degeneration Treatment Trials (CATT), 53% of patients on monthly ranibizumab and 71% on monthly bevacizumab had persistent intra- and/or subretinal fluid at one year, with higher rates in the treat-and-extend arms [1, 2]. Persistent intraretinal fluid and a thicker subretinal tissue complex portend worse visual outcomes [3–5]. Alternate therapies may be of benefit.

Sirolimus, the generic name for rapamycin, is a macrolide compound produced by the bacterium *Streptomyces hygroscopicus.* It has been FDA approved for the prevention of kidney transplant rejection and coronary stent coating. Previously studied in small pilot studies, rapamycin’s immunosuppressive and anti-proliferative qualities, make it an intriguing option for the treatment of exudative AMD [6–8]. In mouse retinal laser photocoagulation models, oral treatment with sirolimus significantly reduced the extent of neovascularization, in a VEGF-independent manner [9]. In senescence-accelerated OXYS rats, systemic sirolimus reduced the severity of retinopathy in a dose-dependent manner [10]. Intravitreal sirolimus was well-tolerated in rabbits and demonstrated retino-choroidal migration supporting its potential use in chorioretinal disease [11, 12]. In human studies, Sirolimus has been used systemically in a 3 person cohort and demonstrated a decreased number of needed intravitreal Avastin injection per month [6]. Finally, intravitreal sirolimus has undergone two large phase III studies in the treatment of non-infectious uveitis and is currently undergoing a third phase III study [13, 14]. Our desire is to build upon previous research and examine a larger cohort in a randomized monotherapy comparison of anti-VEGF treatments and intravitreal sirolimus for persistent, wet AMD.

## Patients/materials and methods

### Study design

This prospective, 6-month, subject-masked trial was conducted at a single site. One-year safety monitoring was performed in a subset of subjects—those who were still in the study when the amendment was approved. Each subject provided written informed consent before enrollment. The study site complied with the Health Insurance Portability and Accountability Act and adhered to the tenets of the Declaration of Helsinki.

### Participants

Eligible patients were recruited from the practice of the principal investigator. Inclusion criteria included an age of 50 years or older, best-corrected visual acuity (VA) measured between 5 and 75 Early Treatment of Diabetic Retinopathy Study (ETDRS) letters (Snellen equivalent 20/32–20/800), presence of choroidal neovascularization secondary to AMD, at least 3 previous intravitreal anti-VEGF injections in the past 5 months, and a lack of response to anti-VEGF therapy defined as continued subretinal or intraretinal fluid with a decrease in central subfield thickness of less than 100 µm since the last injection.

Ocular exclusion criteria included aphakia, a history of pars plana vitrectomy, a history of major ophthalmic surgery in the past 3 months, any ophthalmic surgery within the past 30 days, a history of significant ocular disease other than exudative AMD, uncontrolled ocular hypertension and the presence of significant epiretinal membrane.

### Treatment groups and randomization

Eligible subjects were randomly assigned to one of two treatment arms in a 1:1 ratio. Eyes assigned to group 1 received 440 µg (20 µL) of intravitreal sirolimus at baseline, month 2, and month 4, and sham injections at months 1, 3 and 5. Eyes assigned to group 2 continued their pre-study treatment regimen of either bevacizumab (1.25 mg/0.05 mL) or aflibercept (2 mg/0.05 mL). Bevacizumab injections were repeated at monthly intervals and aflibercept was given at baseline, month 2 and month 4, and sham injections at months 1, 3 and 5.

Rescue criteria for group 1 (sirolimus treatment arm), requiring a switch to anti-VEGF injections, included a 10 ETDRS letter decrease in VA at two consecutive visits, a 15 letter decrease in VA at any visit, a CST increase of 50 µm or more associated with a 5 or more letter decrease in VA, the presence of new hemorrhage, worsening hemorrhage, or at the discretion of the investigator.

### End points and statistical analysis

Primary efficacy measures included the change from baseline in CST and VA. The change in CST was chosen as the primary measure because the inclusion criteria selected patients with treatment resistant disease, for which a change in visual acuity would be less likely. Secondary measures included change in central choroidal neovascularization area, intraretinal fluid area and total subretinal fluid area. The same Heidelberg Spectralis machine was used to obtain OCT images and the automated CST segmentation was verified and manually adjusted as necessary. The central choroidal neovascularization (CNV) lesion area (mm^2^), intraretinal fluid (IRF) area (mm^2^) and subretinal fluid (SRF) areas (mm^2^) were calculated by an independent, masked OCT reader.

Generalized linear models were used to perform a repeated measures analysis to determine if the sirolimus group followed the same trend in the mean primary outcomes over time as compared to the anti-VEGF group. This was performed using the Kenward-Rogers degrees of freedom adjustment which uses all available data in parameter estimation allowing full use of follow-up data as well as allowing a more accurate estimator of the sampling covariance matrix in small samples [15, 16]. It was assumed that both the change in CST and change in VA from baseline followed a linear trend over time and the unstructured covariance was estimated for each model. The change in VA from baseline was truncated at ± 3 standard deviations of the 6-month mean. For the secondary measures only (IRF, SRF and CNV lesion size), p-values were calculated using a simple two-sample T-test, with unequal variance.

## Results

### Baseline characteristics and follow-up

Between April 27th, 2015 and January 8th, 2016, 40 eyes of 40 subjects were enrolled in the study, with 20 eyes assigned to each treatment arm. The baseline characteristics of both groups are summarized in Table 1. The 6-month evaluation was performed in 20 (100%) of the eyes in the anti-VEGF group and 18 (90%) of the eyes in the sirolimus group. During the study, two of the subjects died in the sirolimus group, one from pneumonia and the other from a pulmonary embolism.

**Table 1:** Baseline summary by treatment

Demographic	Anti-VEGF		Sirolimus	
	N = 20		N = 20	
	Mean	SD	Mean	SD
Age at baseline (years)	79.24	8.86	84.46	5.76
Time with Wet AMD (months)	76.95	55.83	54.20	40.43
Total previous intravitreal anti-VEGF injections	36.95	21.87	29.45	22.03
Baseline visual acuity (ETDRS letters)	53.90	12.87	47.10	16.29
Baseline central subfield thickness (µm)	426.90	115.84	492.80	131.14
Baseline choroidal neovascularization (mm^2^)	4.23	3.22	6.55	4.43
Baseline central macular thickness (µm)	362.04	176.21	392.26	200.50

The baseline summary information summarizes the two study populations at their entry into the study. There was no statistical significance shown between our two study groups

### Treatments

Twenty (100%) eyes in the anti-VEGF group met re-treatment criteria at each of the scheduled injection visits. During the course of the study, one patient received 3 aflibercept injections and nineteen subjects received 6 bevacizumab injections. Seventeen (85%) subjects in the sirolimus group met re-treatment criteria at each of the scheduled injection visits and received a total of 3 sirolimus injections. For the subjects who did not complete a total of 3 sirolimus injections, one patient died prior to the second scheduled injection, one patient missed an appointment at the second scheduled injection and one patient was rescued over to anti-VEGF therapy due to the development of anterior uveitis with an elevated intraocular pressure of 36 mmHg at month 2. One patient, after receiving all three sirolimus injections, was rescued to anti-VEGF therapy due to a decline in VA at month 5 (65 letters at baseline, improving to 68 letters at month 4 after 2 sirolimus injections and suddenly declining to 30 at month 5), due to the development of a central retinal artery occlusion.

### Efficacy measures

**Fig. 1:**
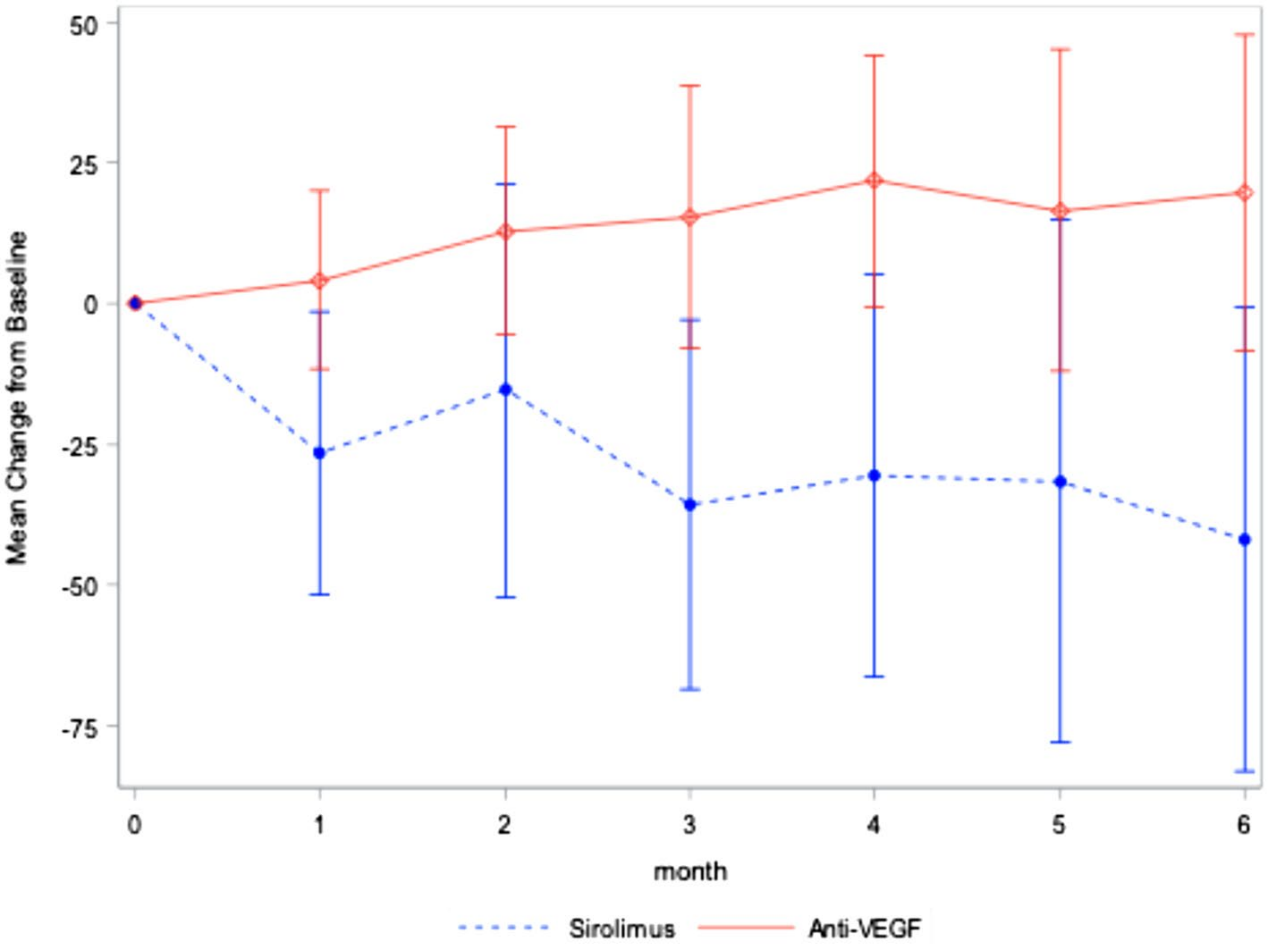
Mean Change in CST from baseline (Observed values, 95% C.I.). This graph depicts the changes in central subfield thickness

**Fig. 2:**
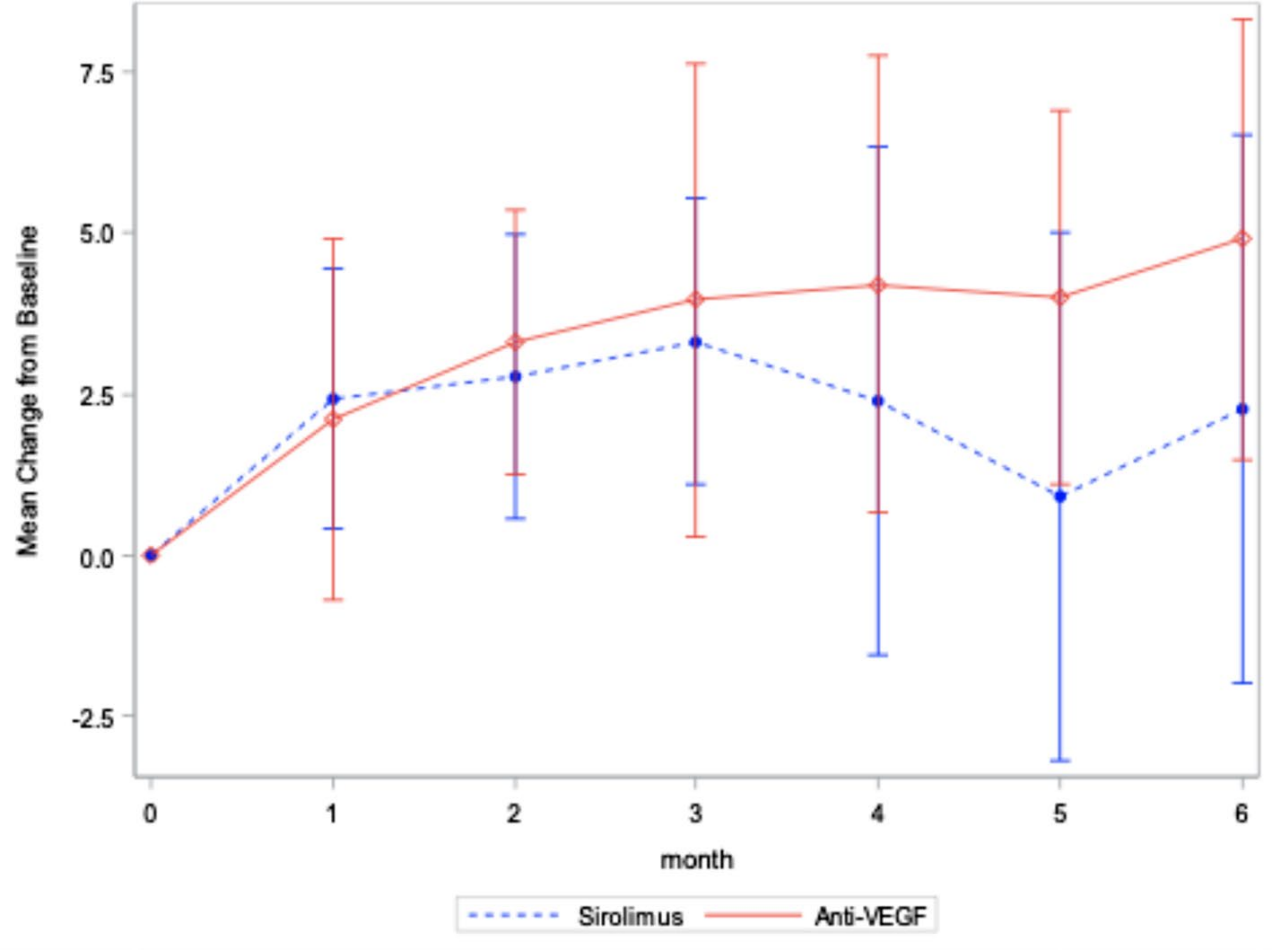
Mean Change in VA from Baseline with 95% C.I. (Observed values). This figure demonstrates the change in CST from baseline for the sirolimus and anti-VEGF groups. There is minimal change in CST for the anti-VEGF group in contrast to the CST decrease in the sirolimus group

The CST outcomes are demonstrated in Fig. 1. At month 6, the mean CST increased by 20 µm in the anti-VEGF group and decreased by 40 µm in the sirolimus group (p = 0.03). After adjusting for confounding variables, the adjusted change in CST is demonstrated in Additional file 1: Fig. S1 and was found to be statistically significant as well. The six-month visual acuity outcomes are demonstrated in Fig. 2. At six months, the mean VA change was + 4.9 ETDRS letters in the anti-VEGF group and + 2.3 letters in the sirolimus group, a difference that was not statistically significant. The adjusted mean change in VA is summarized in Additional file 2: Fig. S2 and the difference was not found to be statistically significant. Covariates with a significant effect on CST outcomes included the treatment, baseline VA and baseline central macular thickness (CMT) and is summarized in Additional file 3: Table S1. At 6 months, the mean change in central intraretinal fluid area was + 0.066 mm^2^ and -0.115 mm^2^ in the anti-VEGF and sirolimus groups, respectively *(p* = *0.75*); the mean change in central choroidal neovascularization area was + 0.310 mm^2^ and -0.538 mm^2^ in the anti-VEGF and sirolimus groups, respectively *(p* = *0.09)*; the mean change in subretinal fluid area was + 0.355 mm^2^ and -0.713 mm^2^ for the anti-VEGF group and sirolimus group, respectively (*p* = *0.04*).

### Safety measures

The safety outcomes for the two groups are summarized in Table 2. There were three serious ocular adverse events in the sirolimus group: a central retinal artery occlusion, a large subretinal hemorrhage, and a subject with anterior uveitis with elevated intraocular pressures. Of the three ocular adverse events, only the anterior uveitis was deemed to be study related. No subjects in the anti-VEGF group developed serious ocular adverse events. The average IOP at month 6 was 14.5 mmHg in the anti-VEGF group and 15.1 mmHg in the sirolimus group and the average change in IOP at all visits was similar between groups.

**Table 2:** Systematic and ocular adverse events

	Sirolimus	Anti-VEGF
Systemic events		
Serious adverse events	3^a^	1^b^
Minor adverse events	3	8
Ocular events		
Serious adverse events	3	0
Minor adverse events	7	7

This table lists the number of adverse events in each group, separated into systematic and ocular adverse events

^a^Two deaths due to pneumonia and pulmonary embolism, and one hospitalization due to a myocardial infection

^b^Hospitalization due to pulmonary embolism

### Study extension

After the study was initiated, and after some subjects had already completed the study, an amendment to the study protocol was approved, to further evaluate safety and efficacy through 12 months. Thirteen subjects participated in this extension, of which only four were previously receiving sirolimus. Efficacy outcomes could not be analyzed due to a low number of subjects. Of note, one of the four subjects receiving sirolimus was rescued to aflibercept due to sterile inflammation at month 9. Another patient receiving sirolimus was rescued to aflibercept due to a subretinal hemorrhage resulting in a VA decline at month 10 (50 ETDRS letters at month 9 versus 43 ETDRS letters at month 10).

### Case review

**Fig. 3:**
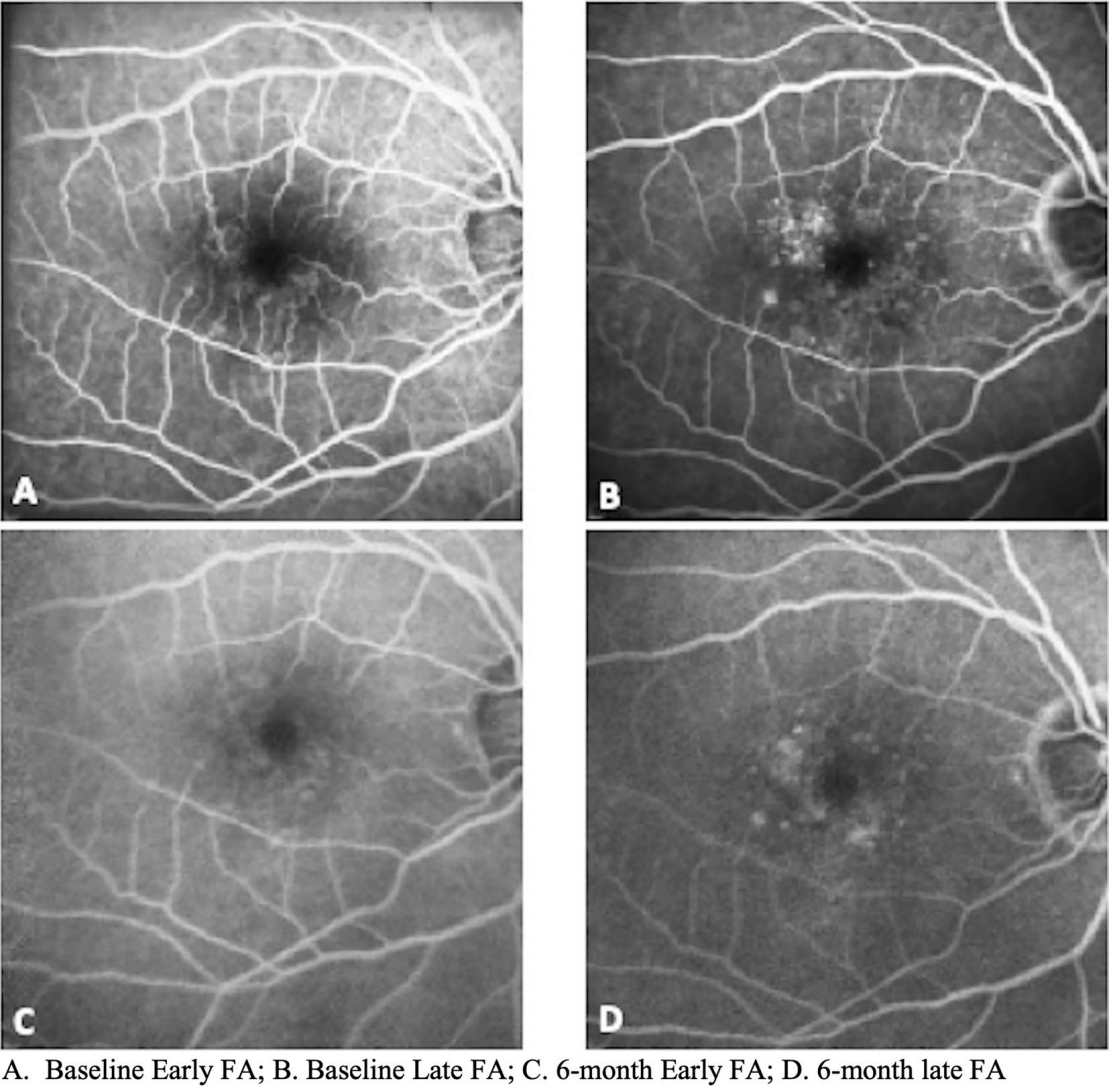
Case 1 Fundus Images. Case 1: Image A is the OCT image of subject 1 at baseline showing subretinal fluid with an adjacent PED. B through F are images at the same location monthly showing gradual anatomic improvement after sirolimus injections every other month. Image G is the 6-month follow-up confirming significantly decreased subretinal fluid

**Fig. 4:**
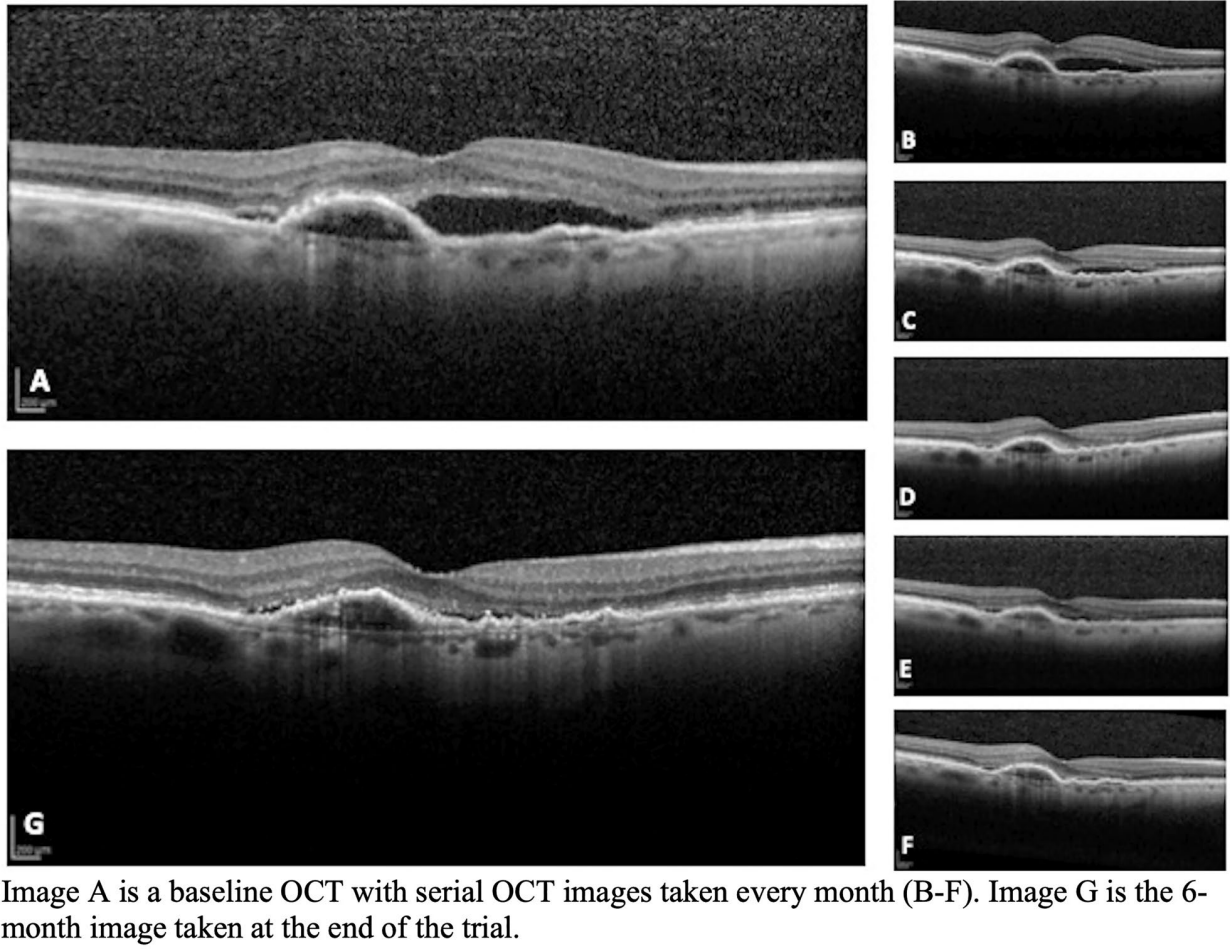
Case 1 OCT Images. Case 1: Image A (early) and B (late) baseline fluorescein angiography. Image B shows late temporal parafoveal hyperfluorescence. Images C (early) and D (late) show 6-month follow-up imaging, where there is decreased hyperfluorescence in the temporal parafoveal area with no new areas of leakage

Case 1: Subject with continued subretinal fluid and decreased vision despite six previous anti-VEGF injections (four bevacizumab and two aflibercept). Baseline and 6-month fluorescein angiography images, Fig. 3, show a significant decrease in leakage after treatment with 3 intravitreal sirolimus injection. Figure 4 shows progressive OCT improvement at each follow up. At baseline (Fig. 4a) the subject presented with CST of 438 µm, visual acuity of 68 ETDRS letters and OCT calculated SRF of 3.71 mm^2^. At 6 months (Fig. 4g), the subject returned with a marked decrease in subretinal fluid and a flattened macula, CST of 283 µm, VA of 70 ETDRS letters, and SRF of 0.18 mm^2^. Case 1 was a subject that met the criterion for entry into the extension protocol. At one year of follow up, with only sirolimus treatments, the subject had a CST of 297 µm and VA of 70 ETDRS letters, with no noted adverse events.

**Fig. 5:**
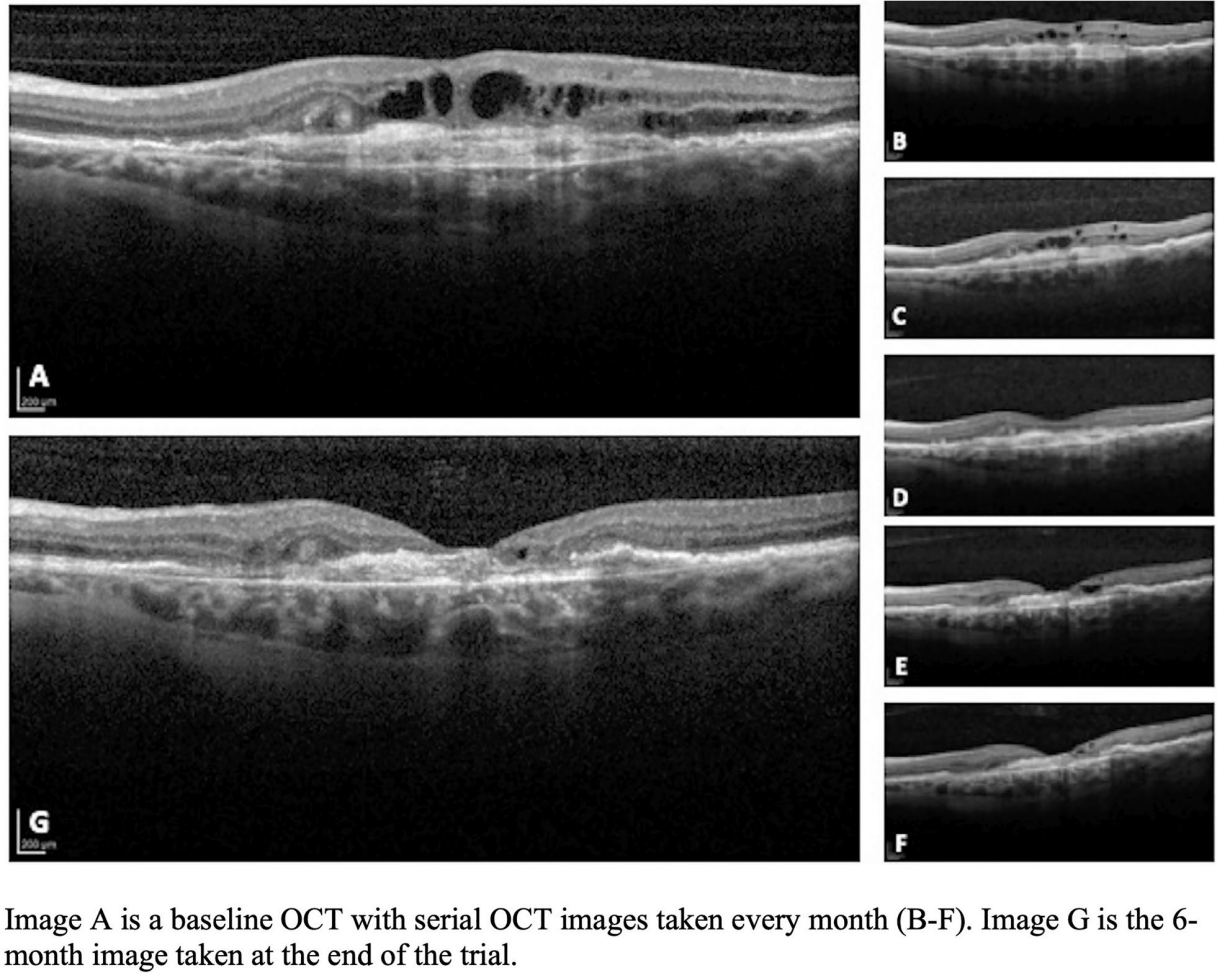
Case 2 OCT Images. Case 2: Image A is the baseline OCT demonstrating significant intraretinal fluid despite 38 previous injections of Anti-VEGF. B through F are imagined at the same location monthly showing a slow resolution of the intraretinal fluid over time. G is the 6-month follow-up showing almost complete resolution of intraretinal fluid and a decreasing size of the subretinal hyperreflective membrane (SHRM). Visual acuity improved 6 letters over the course of the study

Case 2: Subject presented with intraretinal fluid centered on the fovea despite thirty-eight previous anti-VEGF injections over the course of 76 months (all bevacizumab). Figure 5 shows a progression of OCT images at monthly follow up visits; on initial presentation (Fig. 5a) the subject had a CST of 457 µm, VA of 29 ETDRS letters and IRF area of 2.67 mm^2^. At 6-month follow-up (Fig. 5g) the subject is noted to have a marked decrease in central subfield thickness (decrease of 199 µm), and an increase of visual acuity by 6 ETDRS letters. The fluorescein angiogram was clinically improved with resolved leakage (not shown).

## Discussion

**Fig. 6:**
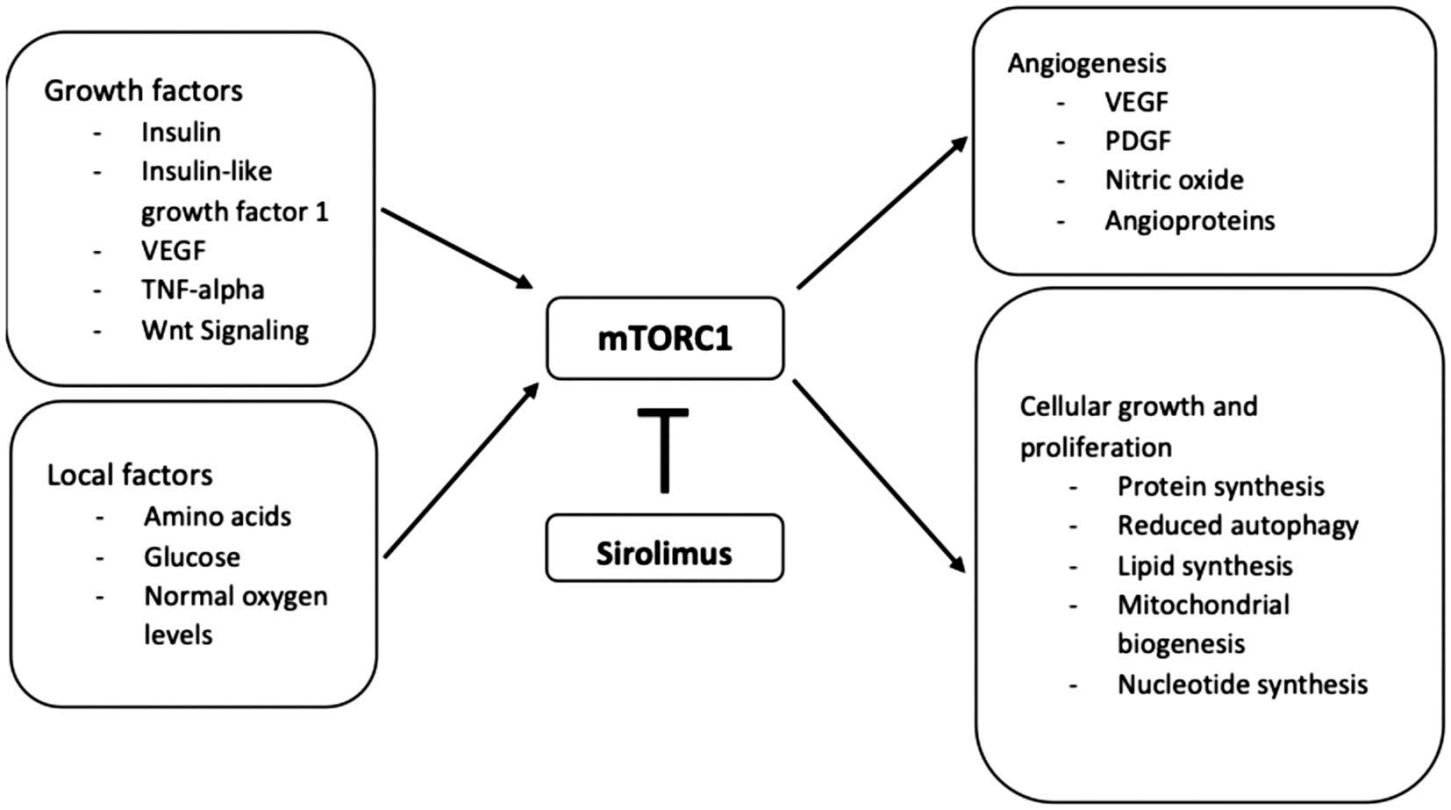
Sirolimus Mechanism of Action Overview. This figure shows a simplified mechanism of action of sirolimus and the potential effect on neovascular AMD pathology [18–21]

Sirolimus targets the mammalian target of rapamycin (mTOR), which is involved in a complex set of cellular functions, including serving as a central regulator of cell metabolism, growth, proliferation, permeability, and survival [17–20]. The mechanism of action of sirolimus is summarized in Fig. 6 [19, 21–23] inhibition of mTOR in AMD is thought to reduce proliferation of blood vessels and dampen the inflammatory cascade [24–26].

A growing list of therapies are under investigation in the management of exudative AMD. Sirolimus is different from most medications in that it potentially targets multiple aspects of AMD pathogenesis earlier in the signaling pathway. Our study is the first to look at intravitreal sirolimus monotherapy as an alternative to anti-VEGF therapy in persistent, exudative AMD. Results suggest an anatomical effectiveness of sirolimus, with a statistically significant decline in CST over a 6-month period. The study population was among the most severe that is encountered in a vitreo-retinal clinic population, with a mean AMD duration of over 5 years and a mean number of 33 anti-VEGF treatments prior to enrollment. Given the severity of disease in these subjects, we did not expect, nor find any significant change in central subfield thickness in the anti-VEGF group. However, we did find a statistically significant CST decrease in the sirolimus treated group, suggesting a therapeutic effect of the drug.

Case 1 and 2 show anatomical changes consistent with the potential efficacy of sirolimus in the treatment of persistent, exudative AMD. In both cases there was a marked decrease in subretinal or intraretinal fluid, a potential positive prognosticator, and a decrease in CST over the 6-month course of treatment. In addition, Case 1 showed the potential of sustained CST decrease over the study extension of 12 months. These cases highlight the potential benefits seen in the study cohort, whom had previously been resistant to further reduction in CST.

A trend towards more ocular adverse events was found in the sirolimus group. By six months, one patient developed uveitis requiring topical steroid drops with resolution over a few weeks. A second subject developed a central retinal artery occlusion, many weeks after sirolimus treatment. Given multiple underlying risk factors, including hypertension, it was felt to be unrelated to the treatment. A third subject, who had previously required chronic anti-VEGF treatment developed a large subretinal hemorrhage. Additionally, two subjects during the one-year extension needed to be rescued from sirolimus to anti-VEGF. We believe that some of the rescued subjects may have needed anti-VEGF treatment in addition to sirolimus due to the rather severe nature of their disease. We plan to present a report of this combination treatment in a future publication.

A study evaluating intravitreal sirolimus for geographic atrophy, noted three cases of sterile inflammation developing among 27 subjects [27]. The association of anterior uveitis with intravitreal sirolimus was also found in a phase III posterior uveitis trial, where a dose-dependent increase in iridocyclitis was found with the use of intravitreal sirolimus. In the 440-µg group, the same dose used in this study, 14.3% of patients developed uveitis and this rate increased to 22.2% in the 880-µg group [13]. The development of anterior uveitis with intravitreal sirolimus warrants further evaluation and potentially changes in formulation or manufacturing to reduce this risk.

Several limitations of this study include a small sample size, short duration of follow-up and variability in treatment history. Additionally, without a prior dose escalation study for exudative AMD, the ideal dose is currently unknown. It is possible, at least for the severe AMD subjects studied, that treatment intervals of less than 8 weeks may be necessary for optimal efficacy.

## Conclusion

Persistent exudative AMD with severe fluid continues to occur in a significant subset of patients undergoing chronic anti-VEGF therapy. We find a statistically significant decrease of CST in this population when treated with sirolimus monotherapy. This pilot study is limited due to the small sample size and chronicity of disease in the study population. A limitation of the Sirolimus group was a higher number of side effects than the anti-VEGF group. Despite improved anatomical outcomes in the study group, these side effects are of concern for the use of Sirolimus as a monotherapy. These preliminary results warrant further investigation due to the positive anatomic effects of Sirolimus, potentially as a combination therapy with anti-VEGF agents, to see if sirolimus has a role in the treatment of persistent exudative AMD.

## Supplementary Information

**Additional file 1: Figure S1.** Adjusted Mean Change in CST by Treatment Group (95% C.I.). This figure demonstrates the adjusted mean change in CST in anti-VEGF and sirolimus groups

**Additional file 2: Figure S2.** Adjusted Mean Change in VA by Treatment Group (95% Confidence Intervals). This figure demonstrates the change in visual acuity for each group over the course of the treatment. This was not found to be statistically significant.

**Additional file 3: Table S1.** Average Change in CST over Time. Repeated Measures ANCOVA, Exploring the Baseline Covariates

## Data Availability

The datasets used and analyzed during the current study are available from the corresponding author on reasonable request.

## Abbreviations

AMD: Age-related macular degeneration
VEGF: Vascular endothelial growth factor
CST: Central subfield thickness
CATT: Comparison of age-related macular degeneration trial
VA: Visual acuity
ETDRS: Early treatment of diabetic retinopathy study
CNV: Choroidal neovascularization
IRF: Intraretinal fluid
SRF: Subretinal fluid
CMT: Central macular thickness
