# Supplementary figures and images for: Intravitreal sirolimus for persistent, exudative age-related macular degeneration: a Pilot Study
Source: Int J Retina Vitreous. 2021 Feb 16;7:11. doi: 10.1186/s40942-021-00281-0 (PMC7885608; doi:10.1186/s40942-021-00281-0)

Adjusted Mean Change in CST

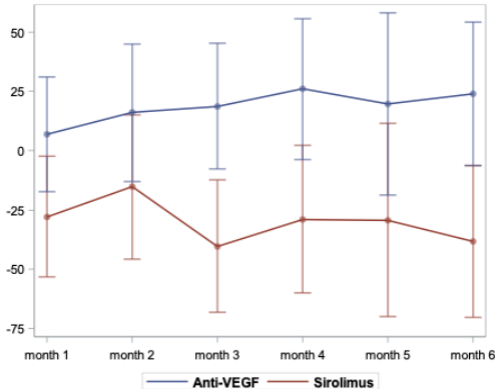

Supplement: Supplementary file 1 — Additional file 1: Figure S1. Adjusted Mean Change in CST by Treatment Group (95% C.I.). This figure demonstrates the adjusted mean change in CST in anti-VEGF and sirolimus groups [file 40942_2021_281_MOESM1_ESM.pdf]

Adjusted Mean Change in VA

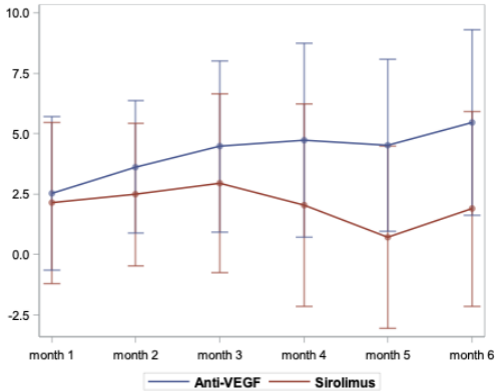

Supplement: Supplementary file 2 — Additional file 2: Figure S2. Adjusted Mean Change in VA by Treatment Group (95% Confidence Intervals). This figure demonstrates the change in visual acuity for each group over the course of the treatment. This was not found to be statistically significant. [file 40942_2021_281_MOESM2_ESM.pdf]
