# Supplementary material for: Intravitreal sirolimus for persistent, exudative age-related macular degeneration: a Pilot Study
Source: Int J Retina Vitreous. 2021 Feb 16;7:11. doi: 10.1186/s40942-021-00281-0 (PMC7885608; doi:10.1186/s40942-021-00281-0)
Supplement: Supplementary file 3 — Additional file 3: Table S1. Average Change in CST over Time. Repeated Measures ANCOVA, Exploring the Baseline Covariates [file 40942_2021_281_MOESM3_ESM.docx]

**Additional Table S1.** Average Change in CST over Time. Repeated Measures ANCOVA, Exploring the Baseline Covariates.

|  | P Value | Estimate | 95% C.I. |
| --- | --- | --- | --- |
| Treatment | 0.0285 | 62.33 | 14.56, 110.09 |
| Month | 0.4535 |  |  |
| Treatment*Month | 0.3337 |  |  |
| Baseline CST | 0.2444 |  |  |
| Baseline VA | 0.0256 | -1.56 | -2.91, -0.21 |
| Age | 0.4988 |  |  |
| Months of Wet AMD at Baseline | 0.9205 |  |  |
| Baseline Presence of Intraretinal Fluid | 0.2419 |  |  |
| Baseline Presence of Subretinal Fluid | 0.1471 |  |  |
| Baseline CMT | 0.0064 | 0.18 | 0.06, 0.31 |
| Baseline CNV | 0.5982 |  |  |

*1. The following covariates were controlled for: baseline CST, baseline VA, age, number of months with wet AMD at baseline, intraretinal fluid present at baseline, subretinal fluid present at baseline, baseline central macular thickness (CMT), and baseline CNV area. The treatment group, time, and treatment by time interaction were evaluated at the 0.05 significance level while adjusting for baseline covariates.*
